# Wave manipulation with magnetically tunable metasurfaces

**DOI:** 10.1038/s41598-017-05625-1

**Published:** 2017-07-14

**Authors:** Hujiang Yang, Tianlin Yu, Qingmin Wang, Ming Lei

**Affiliations:** State Key Laboratory of Information Photonics and Optical Communications and School of Science, Beijing University of Posts and Telecommunications, Beijing, 100876 China

## Abstract

Tunable metasurfaces have emerged as an efficient approach to manipulate the wave propagation. Different from previous work concentrating on electrically tunable mechanisms, here we demonstrate a magnetically tunable metasurface composed of ferrite rods and metallic foils. By tuning the thickness of ferrite rods, metasurfaces with different rod thickness gradients are obtained. The incident wave can propagate through the metasurfaces due to the extraordinary transmission. The deflection angle of the transmission wave is not only influenced by the rod thickness gradient, but also tuned by the applied magnetic field. This approach opens a way for the design of tunable metasurfaces.

## Introduction

Manipulation of electromagnetic (EM) waves (e.g., for phase modulation, light absorption, and imaging applications) has long been restricted by the limited range of EM parameters among natural materials. Electromagnetic metamaterials are composites in which subwavelength features, rather than the natural ones, control the macroscopic electromagnetic properties^1–3^. The unique metamaterial structure makes it possible to achieve considerable electromagnetic response by just using a slab of metamaterial which is much thinner than a wavelength^4^. Metasurface is an ultrathin planar metamaterial comprising artificially designed arrays of subwavelength resonating units, which has attracted significant interests in the optics community^5, 6^. The key point for metasurface is to use resonating unit arrays with subwavelength separation and spatially varying geometric parameters (for example, resonating unit shape, size, orientation) to induce a spatially varying optical response, especially the abrupt changes to the phase and amplitude of the incident wave^7–10^. Therefore, they offer much more opportunities for the control of EM waves than traditional materials.

Recently, several kinds of metasurfaces have been designed and many interesting phenomena have been exploited^11–14^. A metasurface made of “v-shaped” antennas can realize anomalous reflection/refraction of impinging light, which cannot be explained by the classical Snell’s law^15^. Another metasurface perforated with an array of coaxial annular apertures obtained highly efficient beam steering. In spite of the aboved achievements on metasurfaces, the realization of high performance metasurface-based devices still remains a great challenge due to very limited tuning ranges and modulation depths. Great efforts have been paid in developing tunable metasurfaces^16, 17^. Yao *et al*. demonstrated electrically tunable metasurface absorbers with strong light modulation effect by incorporating a metasurface on graphene into an asymmetric Fabry−Perot resonator^18^. Ee *et al*. proposed a mechanically reconfigurable metasurface by fabricating Au nanorod arrays on a stretchable polydimethylsiloxane substrate^19^. The anomalous refraction angle induced by such a metasurface can be adjusted from 11.4° to 14.9° by stretching the substrate by 30%. It is well known that, besides the electrically and mechanically tunable mechanisms, the classic tuning methods also include magnetically tunable one. But, magnetically tunable metasurfaces have not yet been reported so far. Here, we demonstrate a magnetically tunable metasurface that composed of ferrite rods and metallic foils. Combining the ferrite rods with the metallic foils, extraordinary transmission can be realized. The amplitude and phase of the transmission wave can be tuned by the applied magnetic field and also the size of the ferrite rods, which is meaningful for wave manipulation.

## Results

### Design of the magnetically tunable metasurface for wave manipulation

**Figure 1:**
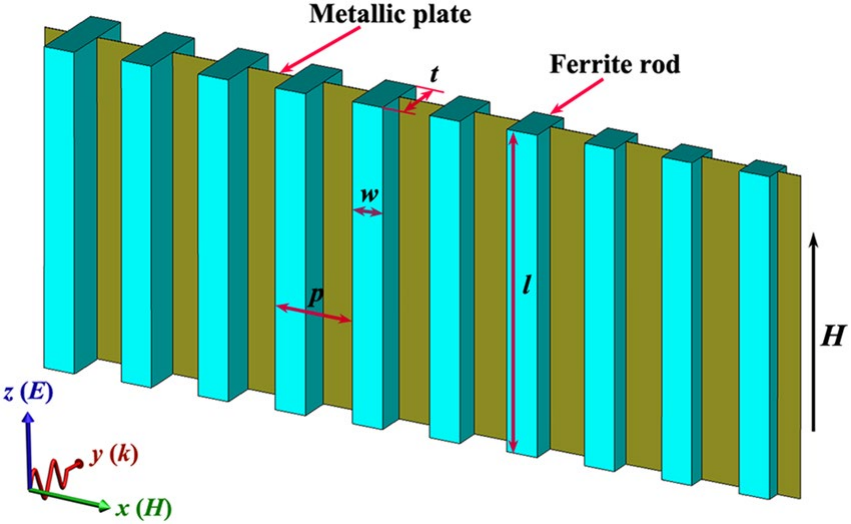
Schematic diagram of the magnetically tunable metasurface.

Figure 1 shows the schematic diagram of the magnetically tunable metasurface for wave manipulation. The metasurface is composed of ferrite rods and metallic foils. The thickness (along *y* direction) of the proposed metasurfaces depends on the thickness of the ferrite rods. The incident wave propagates along the *y* axis, and the electric field and magnetic field are along the *z* and *x* axes, respectively.

**Figure 2:**
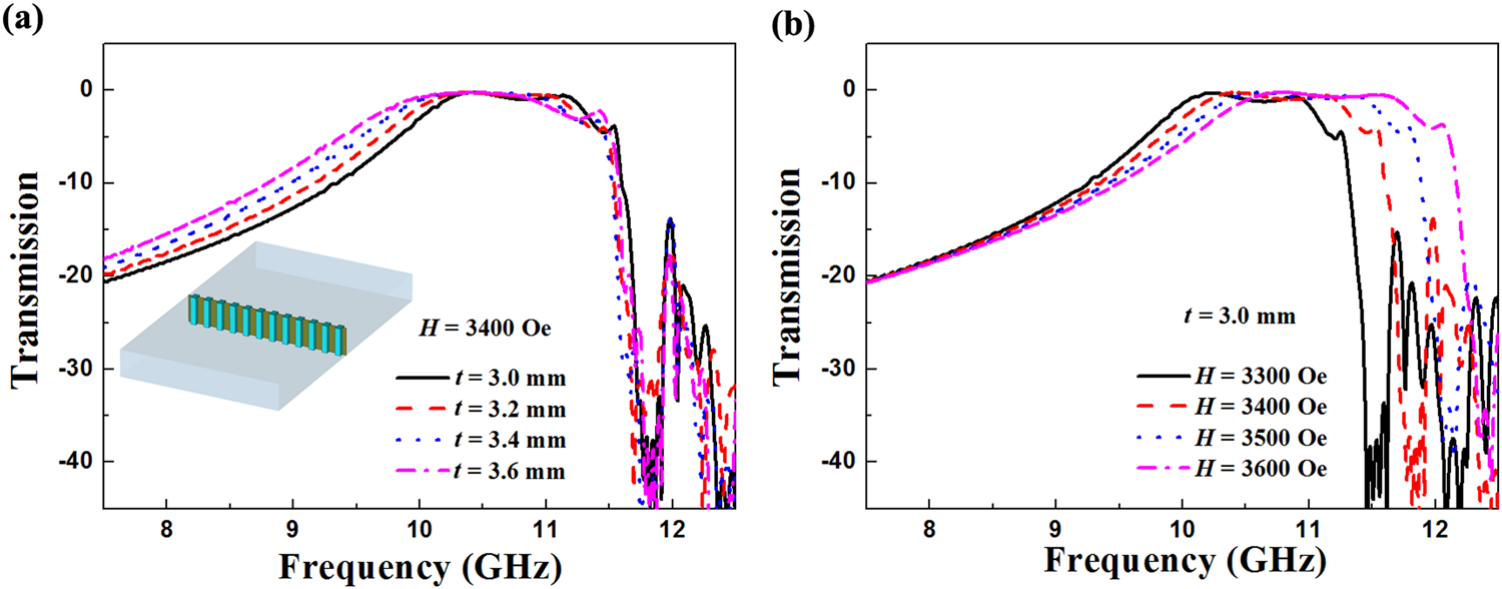
Simulated transmission intensity spectra of the magnetically tunable metasurface. (**a**) The ferrite rods with a series of thicknesses *t*. The inset shows the schematic diagram of the proposed metasurface. (**b**) The ferrite rods under a series of applied magnetic fields *H*.

In order to reveal the magnetically tunable mechanism, firstly the transmission intensity and phase difference of the metasurface with the same ferrite rod thicknesses have been investigated. Figure 2a shows the simulated transmission intensity spectra of the magnetically tunable metasurfaces with a series of ferrite rod thicknesses *t*. The inset shows the schematic diagram of the proposed metasurface. The applied magnetic field was set as 3400 Oe. It is obvious that for all cases, there exists a broadband transparent window when the metasurface has a uniform *t* value. As *t* increases, this transparent window turns to low frequency direction. Figure 2b shows the simulated transmission intensity spectra of the magnetically tunable metasurface under a series of applied magnetic fields *H*. The ferrite rod thickness is set as 3.0 mm. As *H* increases from 3300 Oe to 3600 Oe, the center frequency of the transparent window increases from 10.6 GHz to 11.4 GHz, which shows a magnetically tunable behavior.

**Figure 3:**
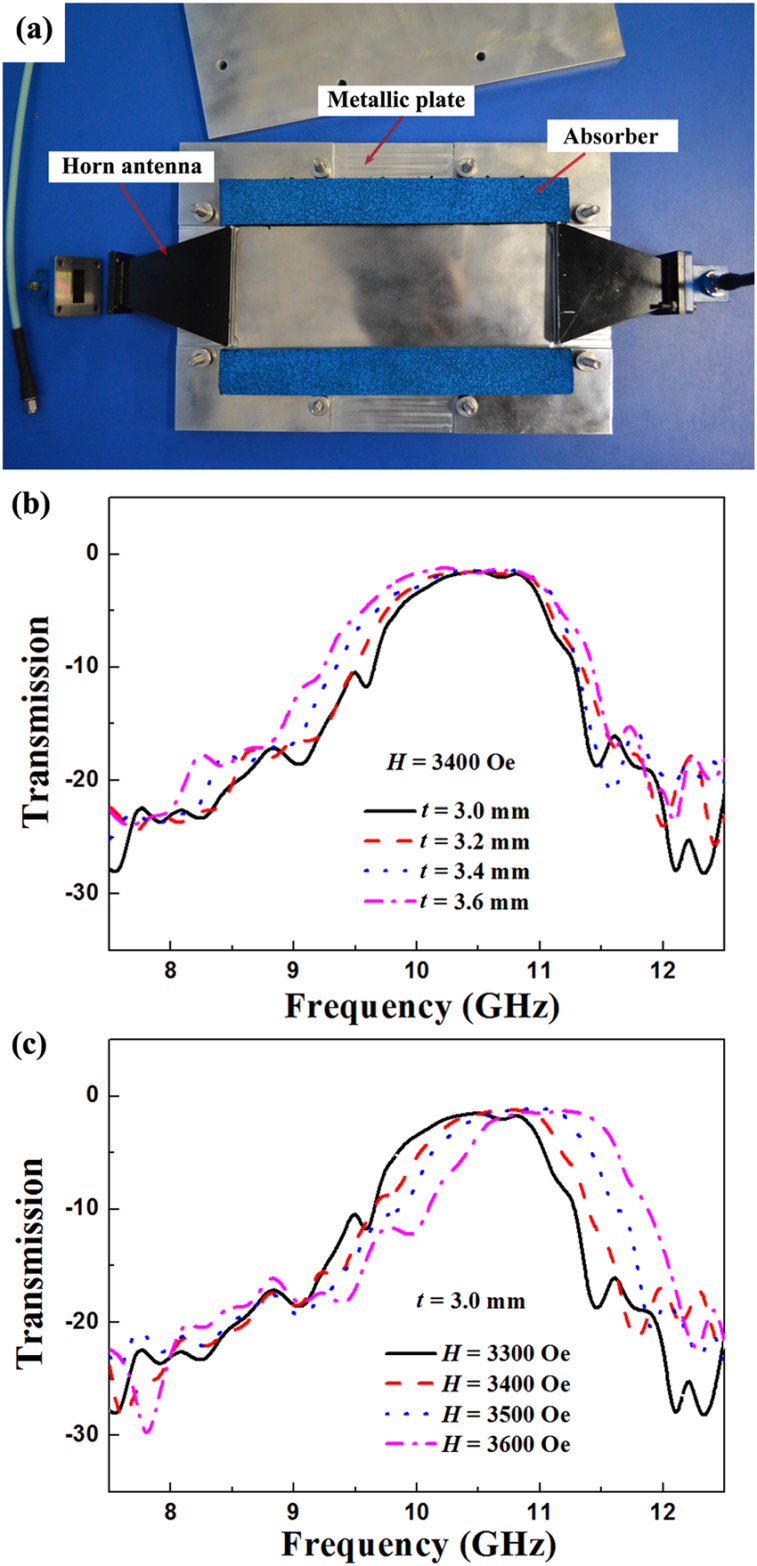
Expeiment demonstrating the magnetically tunable metasurface. (**a**) Photograph of the parallel plate waveguide composed of two horn antennas, two metal plates and absorbers. Measured transmission intensity spectra of the magnetically tunable metasurface. (**b**) The ferrite rods with a series of thicknesses *t*. (**c**) The ferrite rods under a series of applied magnetic fields *H*.

To confirm the above simulated results, transmission intensity spectra of the fabricated magnetically tunable metasurfaces were measured. Figure 3a shows the schematic experimental setup of the metasurfaces placed in the planar waveguide. The bias magnetic field applied in the *z* direction is provided by the electromagnets. Figure 3b shows the measured transmission intensity spectra of the magnetically tunable metasurfaces with a series of ferrite rod thicknesses *t*. The transparent window turns to low frequency direction as *t* increases from 3.0 mm to 3.6 mm. The bandwidth of the transparent window is 1.1 GHz when *t* = 3.6 mm and *H* = 3400 Oe. The measured transmission intensity spectra of the magnetically tunable metasurfaces under a series of applied magnetic fields *H* are shown in Fig. 3c. Due to the material defects and experimental errors, the measured insertion losses are lower than the simulated ones. It can be seen that the center frequency of the transparent window increases from 10.4 GHz to 11.1 GHz with the increasing of the applied magnetic field from 3300 Oe to 3600 Oe, which demonstrates a magnetically tunable property. Obviously, the measured results are in good agreement with the simulated ones.

By interacting with the magnetic field of an electromagnetic wave, ferromagnetic resonance (FMR) can take place in a ferrite under a certain applied magnetic field. The FMR frequency of the ferrite can be expressed by^20^

1 $${f}_{r}=\gamma \sqrt{[H+({N}_{x}-{N}_{z})4\pi {M}_{s}][H+({N}_{y}-{N}_{z})4\pi {M}_{s}]}$$

where *γ* is the gyromagnetic ratio, *H* is the applied magnetic field, *M*_s_ is the saturation magnetization, *N*_x_, *N*_y_, and *N*_z_ are the demagnetization factor at *x*, *y*, and *z* directions, respectively. Based on the above FMR theory, extraordinary transmission can be realized by combining the ferrite rods with the metallic foils^21^. When the FMR takes place at the resonance frequencies, the ferrite rods act as waveguides which can efficiently transmit the electromagnetic wave through the metasurface. According to Eq. (1), the frequency of the passband decreases as the ferrite rod thickness (along *y* direction) increases, and it increases as the applied magnetic field increases. Thus the theoretical prediction is in good agreement with our simulated and experimental results.

**Figure 4:**
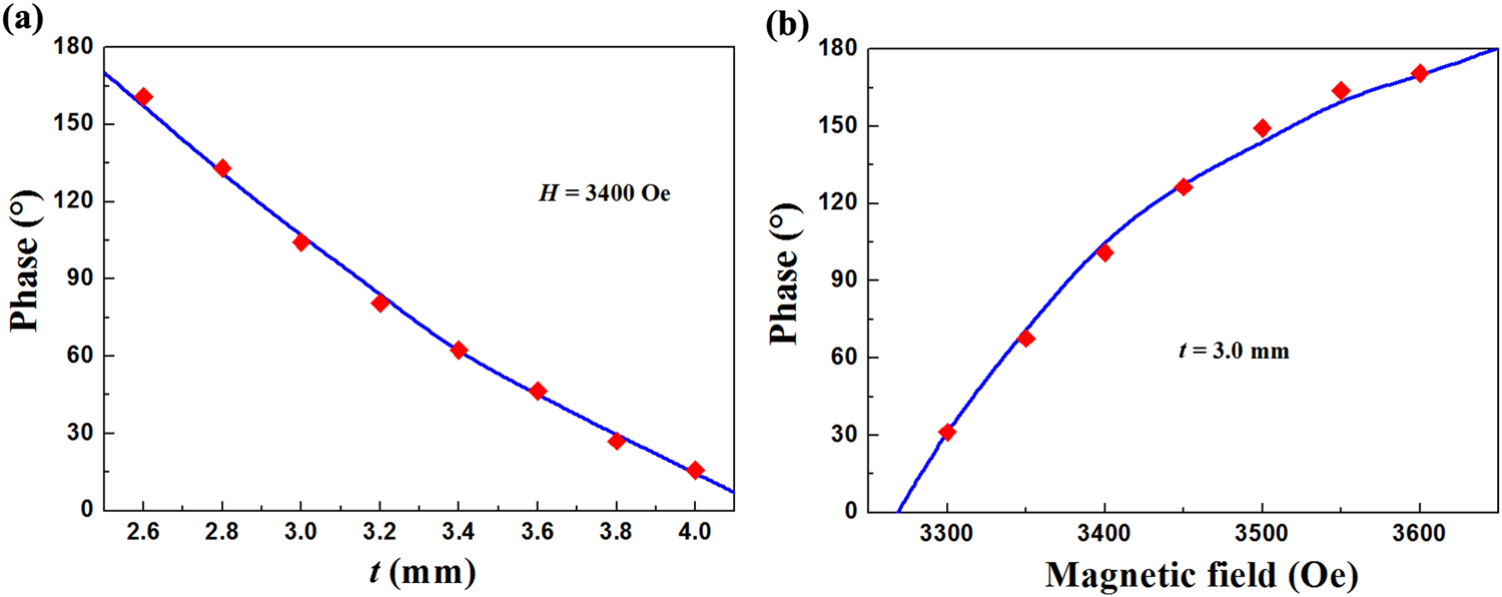
The dependence of phase difference at 11.0 GHz. (**a**) The dependence of phase difference on the ferrite rod thickness *t* and (**b**) the applied magnetic field *H*. The lines and dots represent the simulated and measured results, respectively.

For efficient wave-front manipulation, the capability to create a desired discrete phase gradient at a given operation frequency is of paramount importance^22^. Hence, phase of the proposed metasurfaces are also simulated and measured. All the ferrite rods in one metasurface still have the same thickness value. Before putting the metasurface into the parallel plate waveguide to measure the phase shift, the phase of the incoming wave is normalized to 0° by de-embedding. Figure 4a shows the dependence of phase difference at 11.0 GHz on the ferrite rod thickness *t*. The applied magnetic field is fixed at 3400 Oe. The lines and dots represent the simulated and measured results, respectively. Both the simulated and measured results show that the phase difference varies smoothly. The phase decreases from 161° to 15° as *t* increases from 2.6 mm to 4.0 mm. Figure 3b shows the dependence of phase difference at 11.0 GHz on the applied magnetic field *H*. The ferrite rod thickness is 3.0 mm. It can be seen that the phase increases from 31° to 170° as *H* increases from 3300 Oe to 3600 Oe. Thus Fig. 4 successfully proves that our proposed metasurface can function as an efficient resonant structure, and provides full controlment on the phase of the transmission waves.

### Electric field distributions for magnetically tunable metasurface

**Figure 5:**
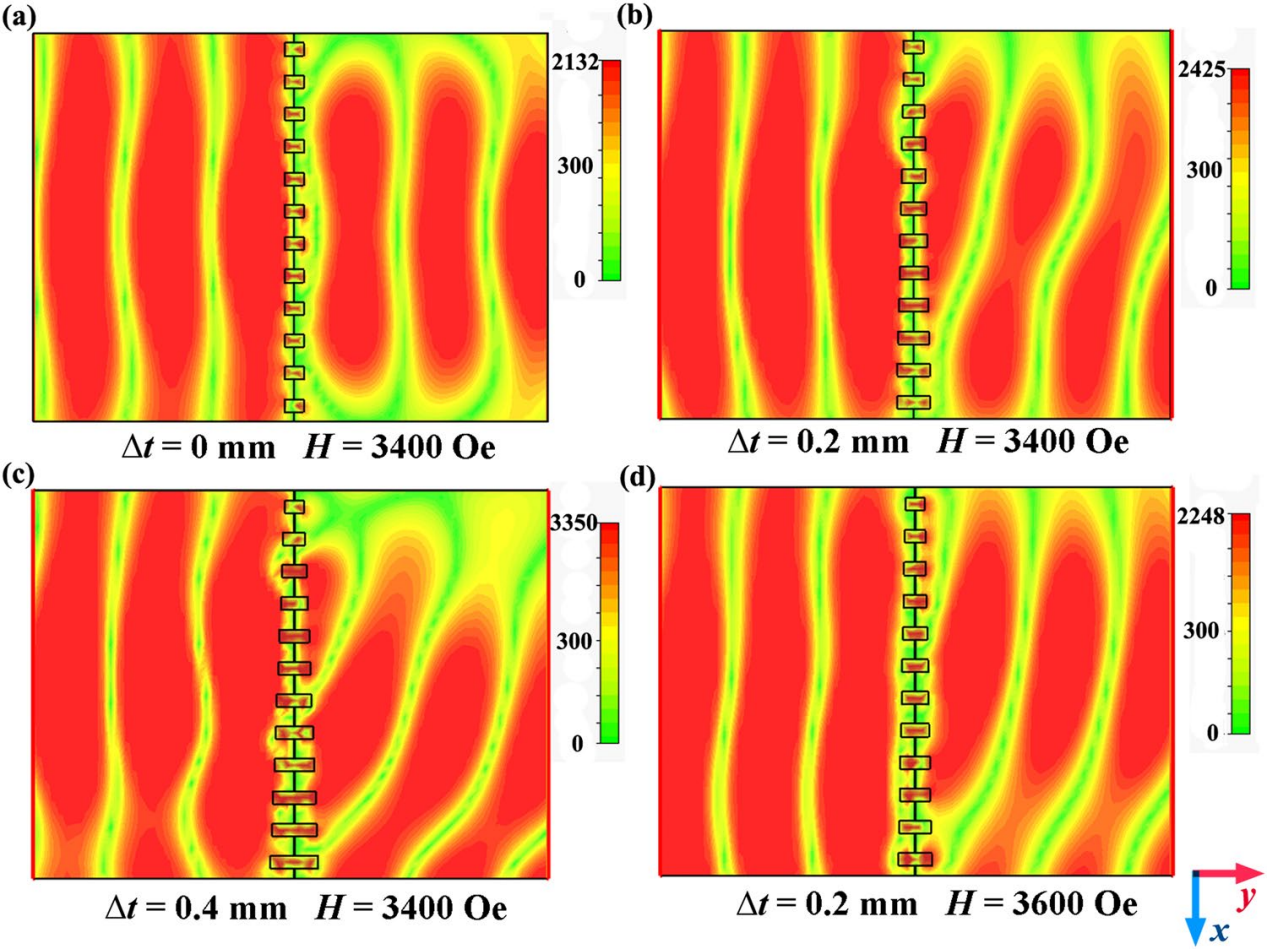
Simulated electric field distributions in the *xy*-plane for a plane wave propagated through the metasurfaces. (**a**) Sample 1 with *H* = 3400 Oe. (**b**) Sample 2 with *H* = 3400 Oe. (**c**) Sample 3 with *H* = 3400 Oe. (**d**) Sample 2 with *H* = 3600 Oe.

The spectra of transmission intensity and phase difference show that wave-front manipulation can be achieved by selecting appropriate values of the ferrite rod thickness. By tuning the thickness of the ferrite rods, phase gradient is assigned along *x* axis on the metasurface, so that the transmission wave can be refracted to a prescribed direction. Three samples with linear phase gradients are used to illustrate this concept. Table 1 presents the ferrite rod thicknesses *t*_n_ of the proposed metasurfaces. The ferrite rod thicknesses for sample 1 have the same value. The thickness gradient *∆t* between two adjacent ferrite rods for samples 2 and 3 are 0.2 mm and 0.4 mm, respectively. To demonstrate the wave manipulation behavior of this design, we simulated the electric field distributions in the *xy*-plane for a plane wave propagated through the metasurfaces by using CST Microwave Studio TM. Figure 5a shows the simulated electric field distribution for sample 1 with *H* = 3400 Oe. Due to *∆t* = 0, the electromagnetic wave propagates through the metasurface without deflection. Figure 5b shows the simulated electric field distribution for sample 2 with *H* = 3400 Oe. The plane wave propagates through the metasurface with a certain deflection angle. Figure 4c shows the simulated electric field distribution for sample 3 with *H* = 3400 Oe. It is obvious that the deflection angle for sample 3 is bigger than that for sample 2. The deflection angle can be expressed by^23^

2 $${\theta }_{t}=\arcsin (\frac{\lambda }{2\pi }\frac{d{\rm{\Phi }}}{dx})$$

where *λ* is wavelength, Φ is the phase difference at a local point brought by the metasurface, *d*Φ/*dx* is the gradient of phase difference along *x* axis. From Eq. (2), the deflection angle *θ*_t_ is influenced by *d*Φ/*dx*. Based on the data shown in Fig. 3a, as *∆t* of sample 3 is bigger than that of sample 2, *d*Φ/*dx* for sample 3 is therefore bigger than that for sample 2, which leads to a larger deflection angle for sample 3. Figure 4d shows the simulated electric field distribution for sample 2 with *H* = 3600 Oe. Based on the data shown in Fig. 3b, the sign of *d*Φ/*dx* for metasurfaces with different *t* is opposite to that for metasurfaces under different *H*. Hence, as *H* increases, the deflection angle decreases, which exhibits a magnetically tunable behavior.

**Table 1:** Ferrite rod thicknesses *t*_n_ of the proposed metasurfaces.

Sample	*t*_1_	*t*_2_	*t*_3_	*t*_4_	*t*_5_	*t*_6_	*t*_7_	*t*_8_	*t*_9_	*t*_10_	*t*_11_	*t*_12_	*∆t*
1	3.0	3.0	3.0	3.0	3.0	3.0	3.0	3.0	3.0	3.0	3.0	3.0	0
2	3.0	3.2	3.4	3.6	3.8	4.0	4.2	4.4	4.6	4.8	5.0	5.2	0.2
3	3.0	3.4	3.8	4.2	4.6	5.0	5.4	5.8	6.2	6.6	7.0	7.4	0.4

## Discussion

In conclusion, a magnetically tunable metasurface composed of ferrite rods and metallic foils has been prepared. Owing to the extraordinary transmission, the metasurface provide a broadband transparent window. The amplitude and phase of the transmission wave decrease as the ferrite rod thickness increases, and increase as the applied magnetic field increases. By tuning the thickness of the ferrite rods, phase gradient is obtained. The deflection angle for the metasurfaces increases as the rod thickness gradient increases. In addition, the deflection angle decreases as the applied magnetic field increases, which exhibits a magnetically tunable behavior. This work provides a way for wave manipulation with a magnetically tunable metasurface.

## Methods

### Sample fabrication

The metallic structure was fabricated by circuit board plotter (LPKF Laser & Electronics Company). The thickness of the PCB substrate and copper layer are 1 mm and 0.035 mm, respectively. The ferrite material used here is commercially available yttrium iron garnet (YIG) ferrite with relative permittivity *ε*_r_ = 14.5, saturation magnetization 4π*M*_s_ = 1950 Oe and linewidth Δ*H* = 10 Oe. The width *w* (along *x* direction) of the ferrite rods is 2 mm. The length *l* of the ferrite rods is 10 mm, which is the same as that of the metallic slits. The lattice period *p* is 5 mm. The thickness *t* (along *y* direction) of the ferrite rods varies gradiently along the *x* direction, which are listed in Table 1. The ferrite rods were bonded with PCB plates by epoxy to form the ferrite-based metasurfaces.

### Microwave measurements

The transmission properties were measured by a microwave measurement system composed of a vector network analyzer, a parallel plate waveguide (shown in Fig. 2a) and an electromagnet, which is the same as that in ref. 24. The upper and lower metal plates form the planar waveguide. The two horns antennas are used to transmit and receive the electromagnetic wave. The size of the final section for the horns is 70 × 10 mm^2^. The width (along *x* direction) and length (along *z* direction) of the proposed metasurfaces is 60 mm and 10 mm, respectively. The thickness (along *y* direction) of the proposed metasurfaces depends on the thickness of the ferrite rods. The incident wave propagates along the *y* axis, and the electric field and magnetic field are along the *z* and *x* axes, respectively. The samples were placed in the parallel plate waveguide and the waveguides were put in the middle of two magnets. The bias magnetic field applied in the *z* direction is provided by the electromagnets. The diameter of the two magnets is 100 mm, which can provide a uniform external magnetic field.

### Simulations

Commercial time-domain package CST Microwave Studio TM was used to carry out the numerical predictions of the transmission intensity, phase difference and electric field distributions. All the simulated parameters of the ferrite rods and metallic foils were the same as those in the experiments. The relative permittivity, saturation magnetization and linewidth of the ferrite rods are set as 14.5, 1950 Oe and 10 Oe. The models in the simulations were set up according to the actual measurement environment. The microwave propagated along *y* direction with the electric field along the z direction and the magnetic field along the *x* direction. The boundaries in the *x* and *z* directions are set as perfect magnetic and perfect electric, respectively.
